# Uremic Toxin-Induced Exosome-like Extracellular Vesicles Contain Enhanced Levels of Sulfated Glycosaminoglycans which Facilitate the Interaction with Very Small Superparamagnetic Iron Oxide Particles

**DOI:** 10.3390/ijms241814253

**Published:** 2023-09-18

**Authors:** Christian Freise, Andreas Zappe, Norbert Löwa, Jörg Schnorr, Kevin Pagel, Frank Wiekhorst, Matthias Taupitz

**Affiliations:** 1 Department of Radiology, Charité—Universitätsmedizin Berlin, Corporate Member of Freie Universität Berlin, Humboldt-Universität zu Berlin, and Berlin Institute of Health, Charitéplatz 1, 10117 Berlin, Germany; joerg.schnorr@charite.de (J.S.); matthias.taupitz@charite.de (M.T.); 2 Department of Biology, Chemistry and Pharmacy, Freie Universität Berlin, Altensteinstraße 23A, 14195 Berlin, Germany; a.zappe@fu-berlin.de (A.Z.); kevin.pagel@fu-berlin.de (K.P.); 3 Metrology for Magnetic Nanoparticles Berlin, Physikalisch-Technische Bundesanstalt Berlin, Abbestr. 2, 10587 Berlin, Germany; norbert.loewa@ptb.de (N.L.); frank.wiekhorst@ptb.de (F.W.)

**Keywords:** extracellular vesicles, uremic toxins, VSOP, glycosaminoglycans, exosomes

## Abstract

Uremic toxins exert pathophysiological effects on cells and tissues, such as the generation of a pro-calcifying subtype of exosome-like extracellular vesicles (EVs) in vascular cells. Little is known about the effects of the toxins on the surface structure of EVs. Thus, we studied the effects of uremic toxins on the abundance of sulfated glycosaminoglycans (GAGs) in EVs, and the implications for binding of ligands such as very small superparamagnetic iron oxide particles (VSOPs) which could be of relevance for radiological EV-imaging. Vascular cells were treated with the uremic toxins NaH_2_PO_4_ and a mixture of urea and indoxyl sulfate. Uremia in rats was induced by adenine feeding. EVs were isolated from culture supernatants and plasma of rats. By proton T1-relaxometry, magnetic particle spectroscopy, and analysis of genes, proteins, and GAG-contents, we analyzed the roles of GAGs in the ligand binding of EVs. By influencing GAG-associated genes in host cells, uremic toxins induced higher GAG contents in EVs, particularly of sulfated chondroitin sulfate and heparan sulfate chains. EVs with high GAG content interacted stronger with VSOPs compared to control ones. This was confirmed by experiments with GAG-depleted EVs from genetically modified CHO cells and with uremic rat-derived EVs. Mechanistically, uremic toxin-induced PI3K/AKT-signaling and expression of the sulfate transporter SLC26A2 in host cells contributed to high GAG contents in EVs. In conclusion, uremic conditions induce enhanced GAG contents in EVs, which entails a stronger interaction with VSOPs. VSOPs might be suitable for radiological imaging of EVs rich in GAGs.

## 1. Introduction

The increased plasma levels of organic or inorganic uremic toxins such as indoxyl sulfate or inorganic phosphate, respectively, could cause renal or cardiovascular injuries such as renal fibrosis, vascular dysfunction or vascular calcification [1,2]. These pathophysiological conditions are often associated with remodeling of the extracellular matrix (ECM) [3], which interacts with cells and soluble factors and therefore regulates tissue homeostasis and various cellular functions [4,5].

We recently found that treatment of endothelial cells (ECs) with a mixture of the uremic toxins urea and indoxyl sulfate (UI) induced the generation of a pro-calcifying uremic subtype of exosome-like extracellular vesicles (EVs) [6]. This complements previous reports showing that the release and the cargo load of EVs is influenced by the surrounding milieu of the donor cells [7,8]. For instance, plasma-derived EV from patients with chronic kidney disease contain altered levels of microRNAs compared to healthy controls. These modified EVs were associated with an altered phenotype of vascular smooth muscle cells (VSMCs) and enhanced vascular calcification in the patients [9].

As only briefly outlined, specific analyses focused on the cargo load and biological function of those EVs, leaving out a closer look at alterations in the surface of the different EV populations [6]. 

EVs are generated from the cell surface as microvesicles (diameter ~100–1000 nm) or as exosomes (~20–150 nm) by fusion of multivesicular bodies with the plasma membrane [10]; thus, they could comprise the same surface glycosylation profile as their host cells [11]. This, in turn, implies a surface-dependent interaction of EVs with various ligands. 

Since surface structures or ECM components in general fulfill multifaceted functional effects and function as a carrier of information in pathologically altered tissue [12]; they are promising targets for a tissue- and disease-specific molecular imaging. Imaging probes that might be useful for radiological imaging of ECM-associated pathophysiological conditions are very small superparamagnetic iron oxide particles (VSOPs). VSOPs are currently used to visualize inflammatory sites in diseases including multiple sclerosis, atherosclerosis, and inflammatory bowel diseases by magnetic resonance imaging (MRI) [13,14]. These studies suggest that VSOPs interact with tissues and cells through the binding of VSOPs to glycosaminoglycans (GAGs) [13,15]. 

In a previous study on imaging experiments on atherosclerotic rabbits, we found a co-localization of EVs with VSOPs in areas rich in GAGs [14], which implies an interaction between EVs, VSOPs and GAGs. 

GAGs are long negatively charged polysaccharides covalently attached to core proteins to form proteoglycans. These proteoglycans are major constituents of the ECM and are essential for life by driving many biological processes, including structural integrity, inflammation, growth and development, and many others. GAGs are assembled in repeating disaccharide units of uronic acid and hexosamine. In heparan sulfate (HS), the uronic acid is either a D-glucuronic acid (GlcA) or a L-iduronic acid (IdoA) coupled to a *N*-acetyl-d-glucosamine (GlcNAc). The HS chain can be modified by sulfation, with an *N*-deacetylase/*N*-sulfotransferase converting the GlcNAc to an *N*-sulfated glucosamine, as well as the addition of sulfates by sulfotransferases at the carbon-2 position of the GlcA/IdoA and the carbon-3 and 6 positions of the glucosamine. Chondroitin sulfate (CS) has a repeating unit of D-glucuronic acid (GlcA) and *N*-acetyl-D-galactosamine (GalNAc), which can be sulfated at the carbon-2 position of the GlcA and the carbon-4 and 6 positions of the GalNAc. Dermatan sulfate is composed of a repeating IdoA-GalNAc unit, which can be modified by sulfation at the carbon-2 position of the IdoA and the carbon-4 and -6 positions of the GalNAc. While the exact alterations in the primary structure of these glycosaminoglycans are currently unidentified, numerous studies have confirmed the occurrence of structures in disease pathology that are absent in healthy tissues.

Continuing our previous study on pathophysiological effects of uremic conditions on EV [6], we therefore aim to analyze the effects of uremic toxins on the abundance and composition of GAGs in isolated EVs and in their host cells. Against the background of a potential future imaging of EVs, we apply ^1^H-T_1_-relaxometry measurements to study the interaction between EVs and VSOPs. Together with the pharmacokinetic properties, the longitudinal T_1_- and transverse T_2_-relaxivities determine the efficacy and the magnetic properties of an MRI in contrast to agent-like VSOPs. Certain factors such as the physiological environment and the interaction of VSOPs with surrounding binding partners—such as EVs—modulate the local electromagnetic field of VSOPs. This provides a direct relationship between a strong interaction of VSOPs with binding partners and decreasing T1-relaxivities of VSOPs and vice versa [16,17,18].

## 2. Results

### 2.1. EVs Derived from Endothelial Cells and Vascular Smooth Muscle Cells Reduce the T1-Relaxivity of VSOPs in a Concentration-Dependent Manner

**Figure 1:**
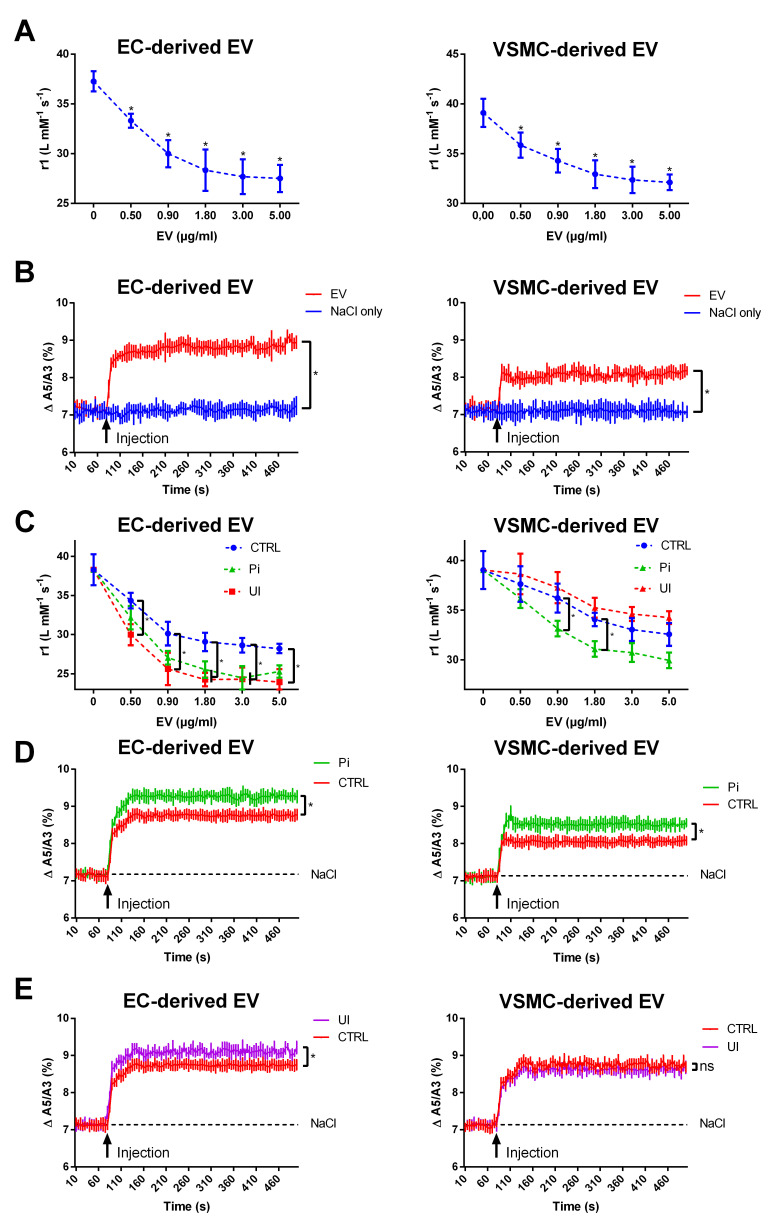
EVs, and in particular uremic toxin-induced EVs, strongly impact the T1 relaxivity and magnetic properties of VSOPs. (**A**) EVs were isolated from ECs or VSMCs. T1 relaxation rates of VSOPs were determined in the presence of rising concentrations of the respective EVs. T1 relaxivites (r1) were determined by linear fitting of T1 relaxation rates in relation to VSOP concentrations. Shown are means ± SD (*n* = 4). (**B**) EVs isolated from ECs or VSMCs were mixed with VSOPs and the resulting effects on the A5/A3 ratio of VSOPs were determined by magnetic particle spectroscopy measurements. Measurement of the vehicle (0.009% NaCl solution) without EVs served as control. Shown are means ± SD (*n* = 3, * *p* < 0.05). (**C**) ECs and VSMCs were treated with Pi or UI for 7 d. EVs were isolated from supernatants. T1 relaxation rates of VSOPs were determined in the presence of rising concentrations of the respective EVs. T1 relaxivites (r1) were determined by linear fitting of T1 relaxation rates in relation to VSOP concentrations. Shown are means ± SD (*n* = 4). (**D**) EV isolated from Pi-treated ECs or VSMCs or (**E**) EVs from UI-treated ECs or VSMCs were injected to VSOPs and the resulting effects on the A5/A3 ratio of VSOPs were determined by magnetic particle spectroscopy measurements. Injection of the vehicle (0.009% NaCl solution) served as control. Shown are means ± SD (*n* = 3, * *p* < 0.05, ^ns^ not significant). Abbr.: A5/A3, dynamic magnetization behavior of VSOPs; EC, endothelial cells; EV, extracellular vesicles; Pi, inorganic phosphate; UI, mixture of urea and indoxyl sulfate; VSMC, vascular smooth muscle cells; VSOP, very small superparamagnetic iron oxide nanoparticles.

First insights into potential interactions between EVs and VSOPs were obtained by T1-relaxivity measurements with VSOPs alone and in combination with rising EV concentrations. EV populations isolated from untreated VSMCs and ECs significantly reduced the T1-relaxivity of VSOPs in a concentration-dependent manner (Figure 1A), which indicates an interaction between EVs and VSOPs. To confirm this interaction, magnetic particle spectroscopy (MPS) measurements were performed. EV populations derived from both cell lines changed the magnetic behavior of VSOPs indicated by an increase in the high harmonic A5/A3 ratio (Figure 1B). Again, the effects of EC-derived EVs were stronger than the effects of VSMC-derived EVs.

### 2.2. EVs Derived from Cells Treated with Uremic Toxins Show a Stronger Interaction with VSOPs in T1 Relaxivity Measurements Compared to Control

Previous data show that the milieu of the host cells could affect the properties of secreted EVs [6]. To experimentally mimic pathophysiological conditions that occur during chronic kidney disease (CKD), ECs and VSMCs were treated with the inorganic uremic toxin phosphate (Pi) or with a mixture of the organic uremic toxins urea and indoxyl sulfate (UI). For ECs, EVs from Pi- and UI-treated cells provoked a stronger reduction in T1 relaxivity of VSOPs than respective EV controls (Figure 1C). A similar effect was observed for EVs from Pi-treated VSMCs (Figure 1D). In contrast, EVs from UI-treated VSMCs had a weaker effect on T1 relaxivity compared to control EVs (Figure 1C). Again, these observations were confirmed by MPS measurements, in that EVs from Pi- treated ECs and VSMCs had a stronger effect on changes in A5/A3 values of VSOPs compared to control (Figure 1D). A similar result was obtained for EVs from UI-treated ECs (Figure 1E). No distinct differences to control on A5/A3 values of VSOPs were observed for effects of EVs from UI-treated VSMCs (Figure 1E).

### 2.3. Treatment of ECs and VSMCs with Pi or UI Alter the Gene Expression Profile of Proteins Being Involved in GAG Homeostasis and Enhances the Amount of GAG in Cells and Isolated EVs

Aiming to investigate the reasons for the different effects of the EV populations, we focused on the abundance of GAGs on the EVs, as these seem to be involved in the interaction between VSOPs and cells (and therefore also EVs) [13]. In the first step, we investigated differences in gene expressions of proteins being involved in cellular GAG metabolism in the host cells. A short description of the respective function of the genes can be found in Table 1.

**Figure 2:**
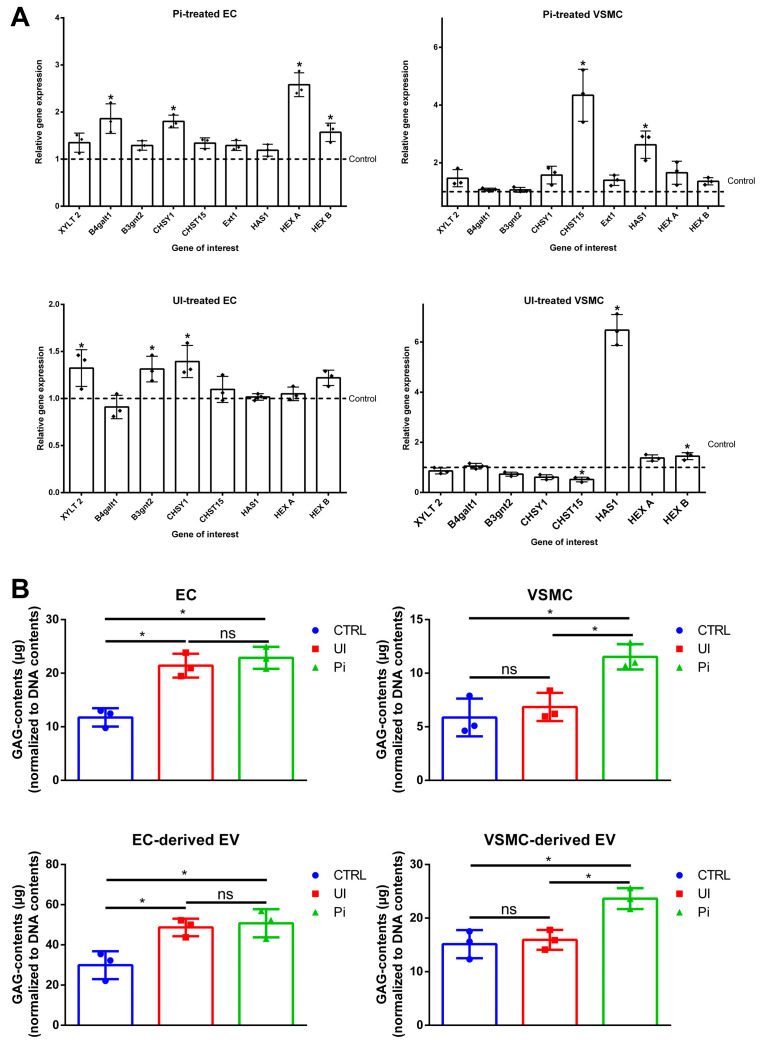
Uremic toxins impact the GAG-relevant gene expression in host cells and modify GAG contents in host cells and isolated EVs. (**A**) ECs and VSMCs were treated for 7 d with Pi (3.5 mM), UI (20 mM UREA, 375 µM IS) or vehicle only as control. Gene expressions were determined by qPCR and were normalized to RPL19 expressions. Shown are means ± SD (*n* = 3). * *p* < 0.05 versus control. (**B**) GAG contents in cells and EVs were quantified using the Blyscan^TM^ assay. Shown are means ± SD (*n* = 3, * *p* < 0.05, ^ns^ not significant). Abbr.: EC, endothelial cells; EV, extracellular vesicles; Pi, inorganic phosphate; RPL19, ribosomal protein L19; GAG, sulfated glycosaminoglycans; UI, mixture of urea and indoxyl sulfate; VSMC, vascular smooth muscle cells.

Pi significantly upregulated genes B4GALT1 (1.86-fold) and CHSY1 (1.80-fold) in ECs (Figure 2A), which, amongst others, are involved in the synthesis and elongation of GAG chains [29,30]. Interestingly, genes HEXA and HEXB, which encode for β-hexosaminidases being involved in the hydrolysis of GAG components [25], were also significantly upregulated (2.58-fold and 1.57-fold, respectively) (Figure 2A).

In Pi-treated VSMCs, expressions of several GAG-specific genes like XYLT2, CHSY1 and HEXA tended to be enhanced; however, only the CHST15 (4.34-fold) gene, which encodes for a 6-O-sulfotransferase involved in the formation of sulfated GalNAc4S6S residues of CS/DS [21], and HAS1 (2.63-fold), which is involved in the synthesis of hyaluronic acid, were significantly upregulated (Figure 2A). 

In ECs treated with UI, gene expressions of XYLT2 (1.32-fold), B3GNT2 (1.31-fold) and CHSY1 (1.39-fold) were significantly upregulated (Figure 2A). In contrast, UI-treated VSMCs comprised a downregulation of CHSY1 (0.61-fold) and CHST15 (0.52-fold) while genes HAS1 (6.48-fold) and HexB (1.45-fold) were significantly upregulated (Figure 2A). 

We subsequently quantified the amounts of GAG in the cells and in the respective isolated EVs using the Blyscan^TM^ Sulfated Glycosaminoglycan Assay kit. Significantly increased GAG contents were measured in Pi- and UI-treated ECs (Figure 2B). In VSMCs, only Pi significantly enhanced GAG contents while UI showed only minor effects (Figure 2B). Like the host cells, isolated EVs from Pi- and UI-treated ECs also comprised significantly higher GAG contents compared to those of EVs derived from supernatants of control cells (Figure 2B). The Pi- and UI-treated VSMC-derived EV populations also contained elevated levels of GAGs; however, only GAGs in Pi-induced EVs were significantly enhanced compared to control (Figure 2B).

### 2.4. Indirect Modulation of GAG Contents in EVs Affects the Interaction between VSOPs and EVs in T1 Relaxivity Measurements

Since GAG homeostasis and/or the quantity of GAGs in host cells is affected by uremic toxins, we next aimed to selectively modulate the GAG composition in the cells to test the implications on the strength of interaction between the respective isolated EVs and VSOPs in T1 relaxivity measurements.

**Figure 3:**
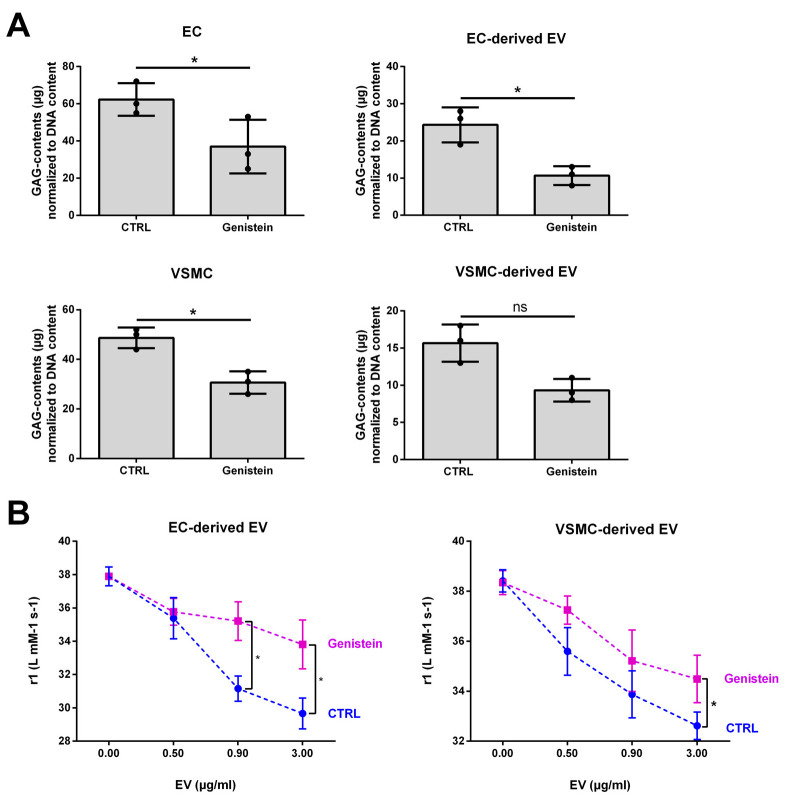
Artificially reduced GAG contents in host cells entail reduced GAG contents in EVs and an attenuated interaction of the EVs with VSOPs. ECs and VSMCs were treated with 15 µM of the inhibitor of cellular GAG synthesis genistein for 3 d or left untreated. EVs were isolated from culture supernatants. (**A**) Contents of GAGs in cells and isolated EVs were determined using the Blyscan^TM^ assay. Shown are means ± SD (*n* = 3). (**B**) Different concentrations of the EVs were mixed with VSOPs and T1 relaxivities were determined by linear fitting of T1 relaxation rates in relation to VSOP concentrations. Shown are means ± SD (*n* = 4, * *p* < 0.05, ^ns^ not significant). Abbr.: EC, endothelial cells; EV, extracellular vesicles; GAG, sulfated glycosaminoglycans; VSMC, vascular smooth muscle cells; VSOP, very small superparamagnetic iron oxide nanoparticles.

We treated VSMCs and ECs with genistein, a known inhibitor of cellular GAG synthesis [31]. Genistein-treated cells as well as isolated EVs comprised reduced GAG contents compared to controls (Figure 3A). In T1 relaxivity experiments, EVs derived from genistein-treated ECs and VSMCs showed an alleviated effect on T1 relaxivity of VSOPs compared to control (Figure 3B).

### 2.5. Treatment of EVs with GAG Lyases Attenuates the Interaction between VSOPs and EVs in T1 Relaxivity Measurements

**Figure 4:**
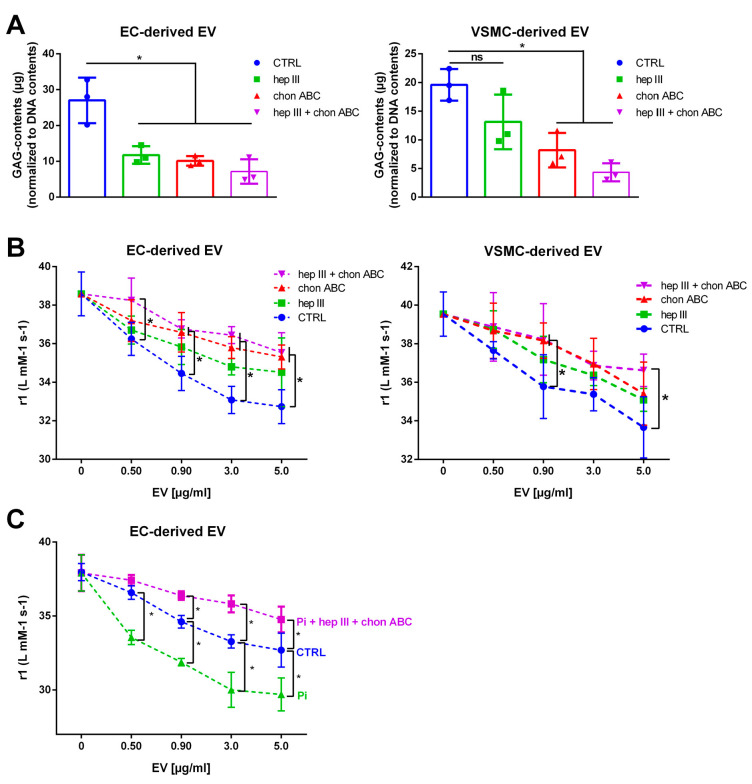
Degradation of GAGs by lyases attenuate the effects of EVs on T1 relaxivities of VSOPs. EVs were incubated with lyases as indicated for 1 h at 37 °C. (**A**) GAG contents in EVs were determined using the Blyscan^TM^ assay. Shown are means ± SD from three independent experiments. (**B**) Different concentrations of control- and lyase-treated EVs were mixed with VSOPs and T1 relaxivities were determined by linear fitting of T1 relaxation rates in relation to VSOP concentrations. Shown are means ± SD (*n* = 4). (**C**) Pi-induced EVs from ECs were treated with lyases as indicated or left untreated. T1 relaxivities were determined as described above. Shown are means ± SD (*n* = 4, * *p* < 0.05, ^ns^ not significant). Abbr.: Chon ABC, chondroitinase ABC; EV, extracellular vesicles; GAG, sulfated glycosaminoglycans; hep III, heparinase III; Pi, inorganic phosphate; VSMC, vascular smooth muscle cells; VSOP, very small superparamagnetic iron oxide nanoparticles.

We next treated isolated EVs directly with heparinase III and/or chondroitinase ABC, which specifically degrade HS and/or CS/DS, respectively [32]. As expected, treatment of EC- and VSMC-derived EVs with the individual enzymes and a combination thereof distinctly reduced the contents of GAGs in the EVs (Figure 4A). In parallel, the effects of the lyase-treated EVs on T1 relaxation of VSOPs were distinctly decreased compared to control. The strongest effects in both EV populations were achieved by treatment with a combination of heparinase III and chondroitinase ABC (Figure 4B).

We next tested this effective combination of both enzymes regarding its effects on the VSOP-T1 relaxation of Pi-induced EVs. Figure 4C shows that the lyase treatment of EC-derived EVs significantly attenuated the effects of the Pi-induced EVs to a level below that of respective control EVs.

### 2.6. EVs from GAG-Depleted Cells Show a Decreased Interaction with VSOPs in T1 Relaxation Studies

To further highlight the functional role of GAGs in the interaction between EVs and VSOPs, we next used EVs isolated from supernatants of commercially available genetically modified CHO cell lines. These are either HS deficient (CRL-2244^TM^) or total GAG deficient due to a defect in xylosyltransferase I (CRL-2242^TM^). The wild-type CHO cell line CCL-61™ served as control. 

**Figure 5:**
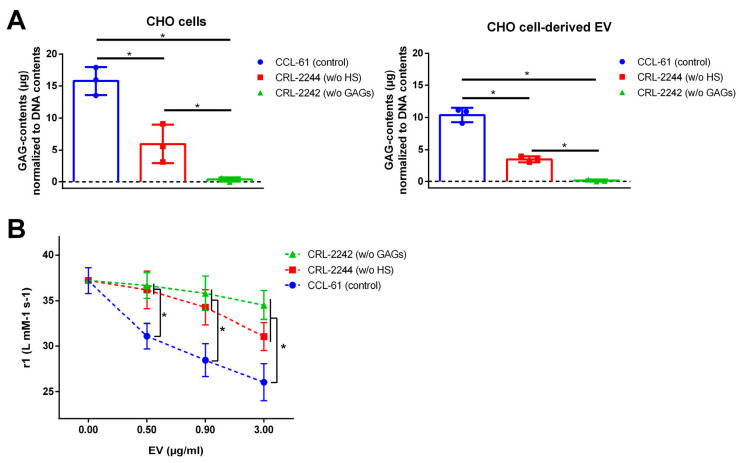
EVs were isolated from different CHO cell lines either with normal GAG contents (CCL-61), with depleted HS (CRL-2244) or without complete GAG expression (CRL-2242). (**A**) GAG contents in cells and isolated EVs were determined using the Blyscan^TM^ assay. Shown are means ± SD from three independent experiments. (**B**) Different concentrations of the different EV populations were mixed with VSOPs and T1 relaxivities were determined by linear fitting of T1 relaxation rates in relation to VSOP concentrations. Shown are means ± SD (*n* = 4, * *p* < 0.05). Abbr.: CHO, immortalized chinese hamster ovary cells; EV, extracellular vesicles; GAG, sulfated glycosaminoglycans; HS, heparan sulfate; VSOP, very small superparamagnetic iron oxide nanoparticles.

First, we quantified GAG contents of cells and EVs. As expected, wild-type CHO cells displayed the highest content of GAGs followed by HS-deficient cells. No GAGs were detectable in CRL-2242 cells (Figure 5A). In the respective isolated EVs, similar patterns for GAG contents were observed (Figure 5A).

Like EVs from ECs and VSMCs, also EVs derived from the control cell line CCl-61™ reduced T1 relaxivity of VSOPs in a concentration-dependent manner (Figure 5B). In comparison, the effects of EVs from HS- and total GAG-deficient cells on T1 relaxivity of VSOPs were significantly reduced for every EV concentration used (Figure 5B).

### 2.7. EVs Isolated from Pi- and UI-Treated ECs and VSMCs Comprise Treatment-Dependent Differences in HS and CS Contents including the Degree of Sulfation

Aiming to move one step further in analysis of treatment-dependent changes in GAGs in EVs, we next investigated the structure and the degree of GAG sulfation in the different EV populations.

**Figure 6:**
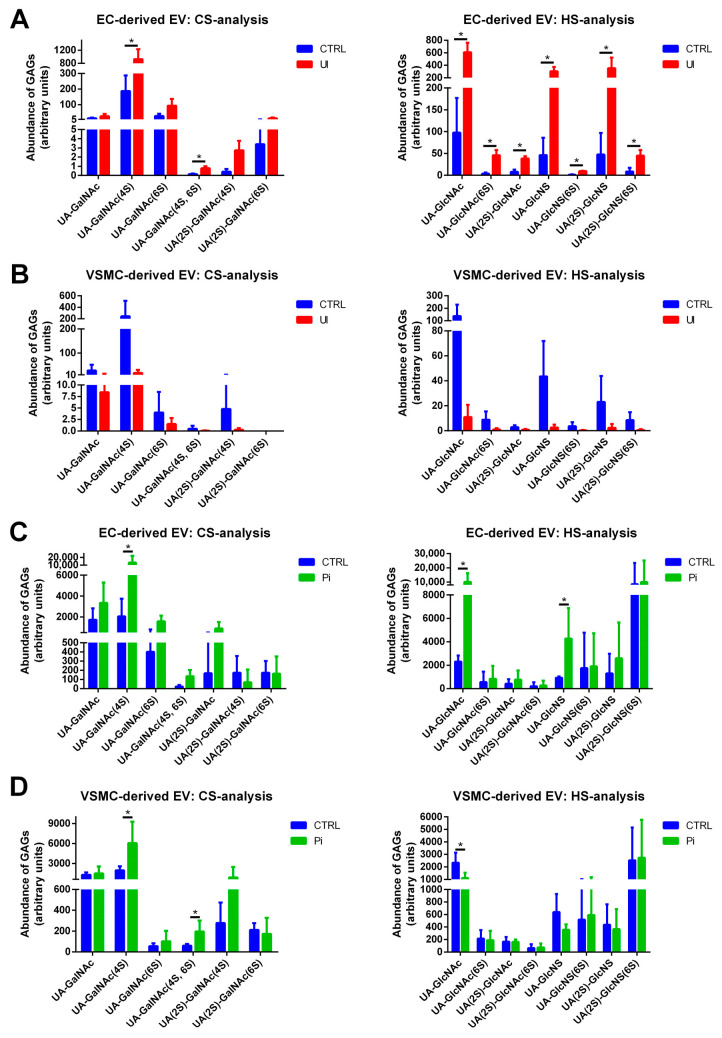
Treatment of host cells with uremic toxins leads to changes in sulfate patterns of GAGs in isolated EVs. (**A**,**B**) ECs and VSMCs were treated with UI for 7 d or left untreated. EVs were isolated from culture supernatants and the analysis of sulfate patterns in GAG oligosaccharides in isolated EVs was performed by UHPLC. Shown are means ± SD (*n* = 4, * *p* < 0.05). (**C**,**D**) ECs and VSMCs were treated with Pi for 7 d or left untreated. EVs were isolated from culture supernatants and the analysis of sulfate patterns in GAG oligosaccharides in isolated EVs was performed by UHPLC. Shown are means ± SD (*n* = 4, * *p* < 0.05). Abbr.: EC, endothelial cells, EV, extracellular vesicles; GAG, glycosaminoglycans; Pi, inorganic phosphate; UHPLC, ultra-high-performance liquid chromatography; UI, mixture of urea and indoxyl sulfate; VSMC, vascular smooth muscle cells.

EVs derived from UI-treated ECs contained distinctly increased overall levels of HS and CS compared to control EVs (Figure 6A). Remarkably, UI-induced EVs comprised a higher degree of 6-O-sulfation in HS and in 4-O-/6-O-sulfation in CS compared to control (Figure 6A). 

In contrast, EVs derived from UI-treated VSMCs displayed slightly decreased amounts of GAGs compared to control (Figure 6B). Also, the degree of sulfation in HS and CS subunits was reduced (Figure 6B).

The treatment of ECs with Pi also provoked higher GAG contents in the respective isolated EVs compared to control. Besides significantly enhanced contents of the chondroitin unit uronic acid-N-acetylgalactosamine (UA-GalNAc)(4S), a trending increase in UA-GalNAc(6S), UA-GalNAc(4S,6S) and UA(2S)-GalNAc units was measured in Pi-treated ECs (Figure 6C). Further, Pi induced significantly higher levels of the HS units uronic acid-N-acetylglucosamine (UA-GlcNAc) and uronic acid-N-sulfated glucosamine (UA-GlcNS) in EC-derived EVs (Figure 6C). 

In VSMCs, Pi also significantly increased the contents of the sulfated chondroitin units UA-GalNAc(4S) and UA-GalNAc(4S,6S) and provoked a trending increase in UA(2S)-GalNAc(4S) compared to control. In contrast, Pi reduced HS (Figure 6D).

### 2.8. The Sulfate Transporter SLC26A2 Is Involved in Uremic Toxin-Induced Formation of GAG-Enriched EVs

**Figure 7:**
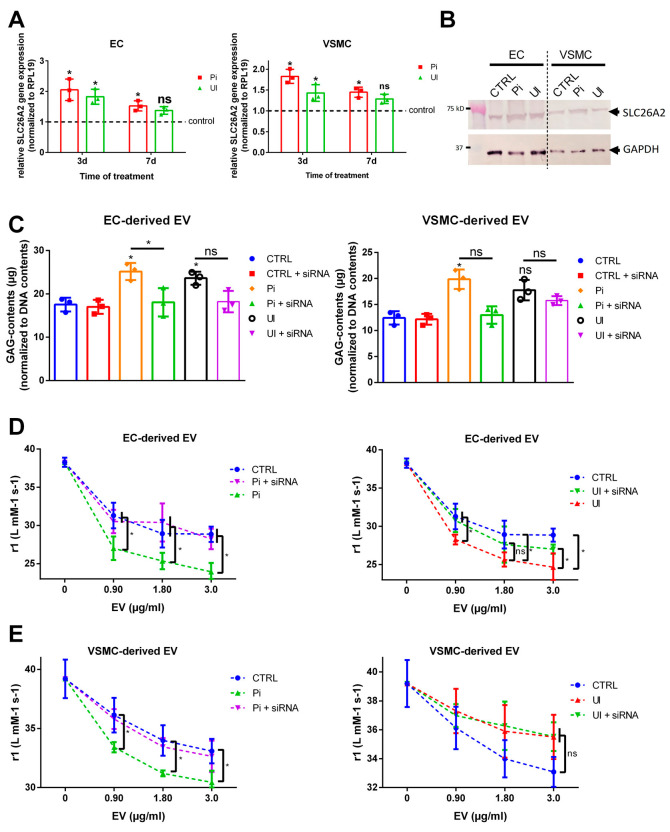
Uremic toxins impact the expression of the sulfate transporter SLC26A2 in host cells which entails altered GAG contents in isolated EVs and a modified EV–VSOP interaction. ECs and VSMCs were treated as indicated. EVs were isolated from culture supernatants. (**A**) Effects of the uremic toxins on relative gene expression of SLC26A2 in the cells were determined by qPCR. Shown are means ± SD (*n* = 3, * *p* < 0.05, ^ns^ not significant). (**B**) Protein expression of SLC26A2 in the cells was determined by Western blot. Shown is one representative blot out of three. (**C**) SCL26A2 was transiently knocked down in the cells by specific siRNA treatment. Effects on GAG contents in isolated EVs were determined using the Blyscan^TM^ assay. Shown are means ± SD (*n* = 3, * *p* < 0.05, ^ns^ not significant). (**D**,**E**) Effects of the SLC26A2 knock-down on the interaction between uremic toxin-induced EVs and VSOPs were determined by T1 relaxivity measurements. Different concentrations of the EVs were mixed with VSOPs and T1 relaxivities were determined by linear fitting of T1 relaxation rates in relation to VSOP concentrations. Shown are means ± SD (*n* = 4, * *p* < 0.05, ^ns^ not significant). Abbr.: EC, endothelial cells, EV, extracellular vesicles; Pi, inorganic phosphate; GAG, sulfated glycosaminoglycans; SLC26A2, solute carrier family 26 member 2; UI, mixture of urea and indoxyl sulfate; VSMC, vascular smooth muscle cells; VSOP, very small superparamagnetic iron oxide nanoparticles.

The data sets indicate that treatment-dependent changes of GAG contents in cells influence the interaction between isolated EVs and VSOPs. We therefore analyzed the involvement of the sulfate transporter SLC26A2, which regulates the sulfation of GAGs [27]. Indeed, treatment with Pi or UI significantly increased gene expressions of SLC26A2 after 3 days in ECs and VSMCs compared to control. After 7 days of treatment, the effects were less pronounced (Figure 7A). In line with the gene expression data, Pi and UI also induced a higher protein expression of SLC26A2 in ECs after 3 days of treatment (Figure 7B). In VSMCs, only Pi induced SLC26A2 protein contents, while UI-treated cells rather showed slightly decreased protein contents of SLC26A2 compared to control (Figure 7B).

We subsequently used specific siRNA to transiently knock down SLC26A2 in ECs and VSMCs by ~80–90% (Supplementary Figure S2) and repeated the previous stimulation experiments with the uremic toxins. A knockdown of SLC26A2 significantly decreased the Pi- and UI-induced generation of GAGs in ECs (Figure 7C). Also, in VSMCs, the depletion of SLC26A2 blocked the Pi-induced formation of GAGs (Figure 7C). As expected from our initial experiments, UI-treated VSMCs comprised no significantly enhanced GAG contents compared to control and the depletion of SLC26A2 had no significant effects (Figure 7C).

In subsequent relaxivity experiments, EVs isolated from SLC26A2-depleted ECs exerted significantly attenuated effects on T1 relaxivity of VSOPs compared to control EVs from Pi- and UI-treated cells with normal SLC26A2 protein expression (Figure 7D). In VSMCs, only the Pi-induced effects of EVs on T1 relaxivity of VSOPs were significantly reduced compared to control, while the effects of EVs from UI-treated VSMCs were not affected by decreased SLC26A2 contents (Figure 7E). As expected from similar GAG contents between the control group and the control+siRNA group (Figure 7C), treatment of cells with siRNA in the absence of uremic toxins had no impact on the effects of isolated EVs on the relaxivity of VSOPs compared to control (Supplementary Figure S3).

### 2.9. Uremic Toxin-Induced Upregulation of SLC26A2 Involves PI3K/AKT Signaling

**Figure 8:**
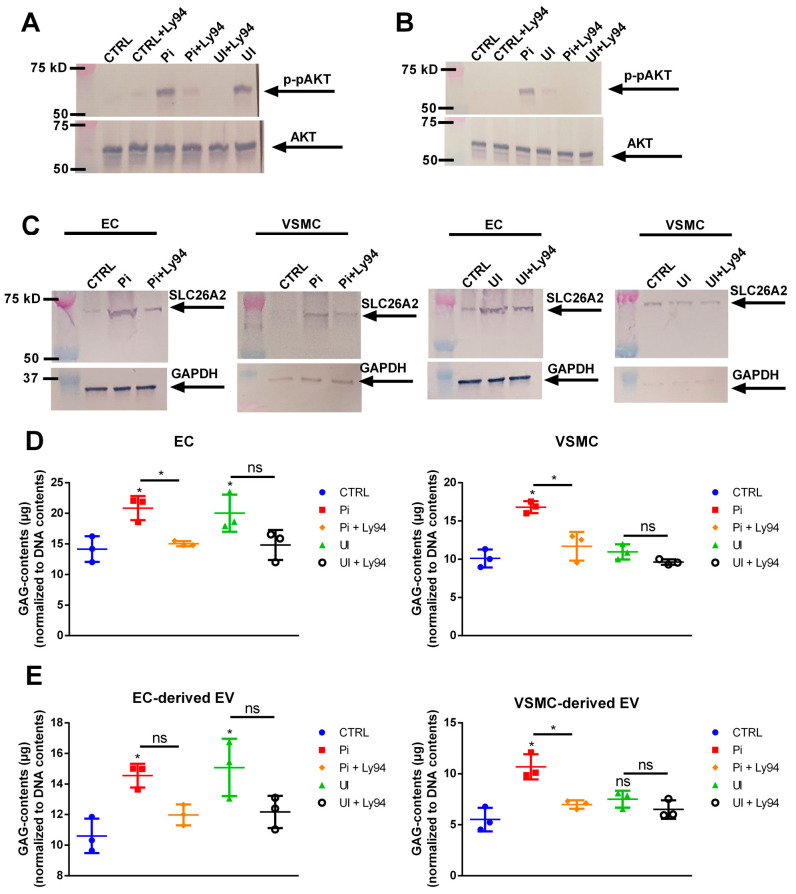
Functional role of PI3K/AKT-signaling for GAG contents and binding properties of EVs. (**A**) ECs and (**B**) VSMCs were treated with Pi or UI for 15 min or left untreated with or without the presence of the PI3K/AKT-inhibitor Ly294002. Protein expression and phosphorylation of AKT was determined by Western blot. Shown are representative blots of three independent experiments. (**C**) ECs and VSMCs were treated for 3 d with Pi or UI with or without the presence of Ly294002. Protein expressions were determined by Western blot. Shown are representative blots of three independent experiments. (**D**,**E**) ECs and VSMCs were treated 3 d with Pi or UI with or without the presence of Ly294002. EVs were isolated from culture supernatants. Treatment-dependent effects on GAG contents in cells and EVs were determined using the Blyscan^TM^ assay. Shown are means ± SD (*n* = 3, * *p* < 0.05, ^ns^ not significant). Abbr.: EC, endothelial cells, EV, extracellular vesicles; GAPDH, glyceraldehyde 3-phosphate dehydrogenase; Ly29, Ly294002—PI3K/AKT-inhibitor; Pi, inorganic phosphate; GAG, sulfated glycosaminoglycans; SLC26A2, solute carrier family 26 member 2; UI, mixture of urea and indoxyl sulfate; VSMC, vascular smooth muscle cells.

Activation of PI3K/AKT signaling is obligatory for the function of SLC26A2 [27]. Hence, we investigated if the observed effects of Pi and UI involve the activation of this pathway. A short treatment of 15 min with Pi and UI led to enhanced phosphorylation of AKT in ECs (Figure 8A) and VSMCs (Figure 8B). As expected, the effects of the uremic toxins on phosphorylation of AKT could be blocked be the presence of the PI3K/AKT pathway inhibitor Ly294002 (Ly29) (Figure 8A,B). Further, the inhibition of AKT phosphorylation by Ly29 reduced the Pi-induced protein expression of SLC26A2 in ECs and VSMCs (Figure 8C). In line with this, the presence of Ly29 during the stimulation with uremic toxins led to distinctly reduced amounts of GAGs in ECs and VSMCs (Figure 8D) and in their respective isolated EVs (Figure 8E). 

### 2.10. EVs from Plasma of Uremic Rats also Reduce T1 Relaxivity of VSOPs

**Figure 9:**
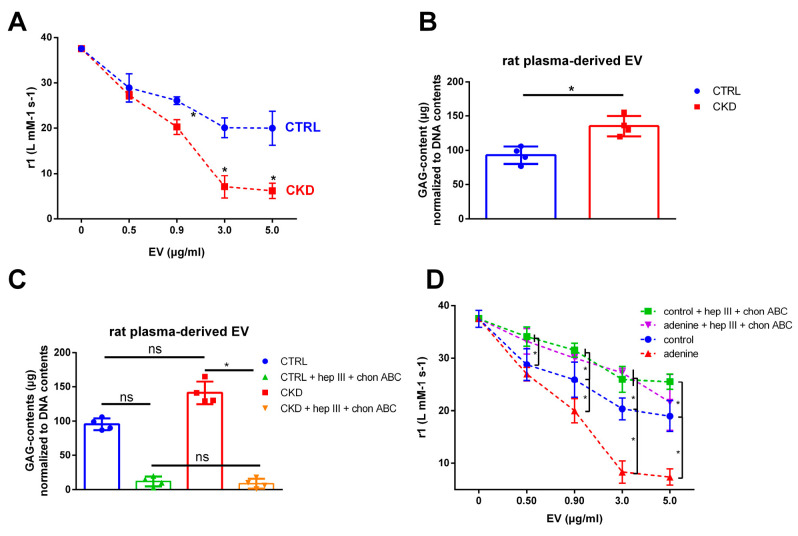
EVs from plasma of uremic rats contain more GAGs and interact stronger with VSOPs compared to control EVs. EVs were isolated from the plasma of uremic rats which were fed adenine or from healthy control animals. (**A**) Different concentrations of EVs were mixed with VSOPs and T1 relaxivities were determined by linear fitting of T1 relaxation rates in relation to VSOP concentrations. Shown are means ± SD (*n* = 4). (**B**) Contents of GAGs in isolated EVs were determined using the Blyscan^TM^ assay. Shown are means ± SD (*n* = 3). (**C**) Isolated EVs were treated with lyases as indicated for 1 h and resulting GAG contents were determined using the Blyscan^TM^-assay. Shown are means ± SD (*n* = 3). (**D**) Effects of the lyases on effects of the EVs on VSOPs were determined by T1 relaxivity measurements. Shown are means ± SD (n = 4). * *p* < 0.05, ^ns^ not significant. Abbr.: Chon ABC, chondroitinase ABC; EV, extracellular vesicles; hep III, heparinase III; GAG, sulfated glycosaminoglycans; VSOP, very small superparamagnetic iron oxide nanoparticles.

In the first approach to translate our in vitro studies to in vivo, isolated EVs from the plasma of control rats and rats with adenine-induced CKD were applied in T1 relaxivity measurements as well. Both EV populations decreased the T1 relaxivity of VSOPs (Figure 9A). In concordance with the in vitro data, the effects of the uremic EV population were significantly stronger than the effects of the control EVs (Figure 9A). These effects were accompanied by higher contents of GAGs in the uremic EVs compared to the control EVs (Figure 9B).

Like for the in vitro-derived EVs, a combined treatment with the lyases hep III and chon ABC distinctly decreased GAG contents in the EVs (Figure 9C) accompanied by a reduced interaction between the EVs and VSOPs in T1 relaxivity measurements (Figure 9D).

## 3. Discussion

Here, we provide evidence that exosome-like EVs that were isolated from supernatants of ECs or VSMCs as host cells interact with the imaging probe VSOPs. This interaction depends on the presence of GAGs on the EVs. Pathophysiological conditions such as the exposure of cells with uremic toxins influence the GAG composition of cells and EVs, thereby also influencing the interaction of EVs with VSOPs. This might enable VSOPs as imaging probes to study EVs and GAG-associated pathophysiological conditions by radiological imaging.

Recent own studies indicated that EVs co-localized with the areas of GAGs and VSOPs in atherosclerotic plaques in rabbits [14], suggesting EVs to be suitable imaging targets for VSOPs. One central aim of this study was therefore to gain insights into the possible interaction between EVs and VSOPs. Indeed, we initially found that exosome-like EVs isolated from ECs and VSMCs dose dependently decreased the T1 relaxivity of VSOPs. Together with the obtained data from the MPS measurements, this indicates a direct interaction between VSOPs and EVs, which presumably leads to increased agglomeration of VSOP cores. 

The T1 relaxivity measurements were used as a surrogate measurement for direct binding studies between EVs and VSOP. The EV-mediated effects on VSOP relaxivity can be explained by a process called transchelation [33]. The negatively charged citrate coating of VSOPs can be replaced by negatively charged surface structures of potential binding partners. The present study shows that EVs contain negatively charged GAGs which could interact with the cationic iron oxide core of VSOPs. These interactions with neighbored molecules decrease VSOP mobility, thereby modulating the local magnetic field of VSOPs and subsequently causing diminished T1 relaxivity values for VSOPs [14,34,35]. The T1 relaxivity values therefore provide a direct relationship between the interactions of VSOPs with binding partners such as EVs. 

This is in line with previous studies showing that VSOPs bind to negatively charged GAGs on cell surfaces [14,34]. This was reflected by changes in the magnetization of VSOPs as assessed by MPS measurements, e.g., during cellular uptake of VSOPs [36]. Here-observed EV-mediated alterations of the magnetic properties of the VSOPs, indicated by an altered A5/A3 ratio after contact with EVs, therefore support our assumption of a direct interaction between VSOPs and EVs, presumably via transchelation. 

Further experiments revealed that the GAG composition and thus the strength of this interaction varies between different EVs and depends on the origin of the EVs. Treatment with the uremic toxins led to an upregulation of genes relevant for the metabolism of GAGs in the host cells. Amongst others, Pi induced the GAG chain synthesizing enzymes B4GLAT1 and CHSY1 as well as the GAG-degrading enzyme HEXA in ECs. In VSMCs, the sulfotransferase CHST15 and HAS1, one out of three isoenzymes responsible for cellular hyaluronan synthesis, were significantly upregulated. This entailed subsequent alterations of GAG contents in the isolated EVs as well. The experimental uremic conditions therefore indirectly influence the interaction of EVs with VSOPs in relaxivity and MPS measurements. 

Other studies demonstrated that pathophysiological conditions such as inflammation can alter the synthesis and sulfation of GAGs as well and, thus, alter the binding of VSOPs to the inflamed tissues or alter the uptake by, e.g., immune cells [13]. For instance, TNF-α induces B4GALT1 in HUVECs [37] and TGF-β induces CHSY1 expression in VSMC [38].

Further own experiments supported the relevance of the toxin-mediated changes in GAG composition for the interaction between EVs and VSOPs. The inhibition of cellular GAG synthesis by genistein reduced GAG contents in cells and EVs and led to attenuated effects of the respective EVs on VSOP-T1 relaxivity. Similar attenuating effects on VSOP-T1 relaxivity were obtained after the incubation of isolated EVs with GAG-degrading lyases. A combination of chondroitinases and heparinases provoked the strongest effects, which confirms the contribution of sulfated CS and HS to the interaction between EVs and VSOPs. 

The functional relationship between GAG contents and the interaction between VSOPs and EVs was further proved by experiments using isolating EVs from supernatants of GAG-deficient CHO cells [39]. In line with previous studies [40], EVs from HS-deficient and total GAG-deficient cells comprised significantly decreased GAG contents and decreased interactions with VSOPs compared to EVs from CHO control cells.

Our hitherto data indicated that the quantity and presumably also the structure of GAGs in cells and EVs are influenced under pathophysiological conditions in our cell culture models, namely elevated levels of organic and inorganic uremic toxins in the culture medium. This complements other studies showing, e.g., an up to 50-fold increase in 6-O-sulfated CS in gastrointestinal carcinoma [41] or elevated 6-O-sulfated and 4-O-, 6-O-disulfated CS structures in ovarian cancer [42,43]. 

Our data confirmed that the experimental uremic conditions provoked significant changes in total GAG contents and GAG fine structures in isolated EVs, including altered degrees of 6-O-sulfation in HS and 4-O-/6-O-sulfation in CS. These data complement the gene expression data in that the Pi-induced upregulation of the sulfotransferase CHST15 in VSMCs, which transfers sulfate to Position 6 of GalNAc4S residues, is reflected by higher contents of sulfated GalNAc4S6S residues in Pi-treated VSMCs. In this context, previous data show significantly increased GAG-contents in arteries of uremic rats [44], which supports the fact that uremic conditions modify GAGs in the cardiovascular system. 

Aiming for underlying mechanisms of the uremic toxin-induced alterations of GAG sulfation in cells and EVs, we found that primarily Pi and to a lesser extent UI induced the expression of SLC26A2 in ECs and VSMCs. A known function of SLC26A2 is the transport of sulfate into chondrocytes to maintain adequate sulfation of proteoglycans [45]. Amongst others, mutations of this sulfate transporter gene are associated with chondrodysplasias [46]. Its transient knockdown in ECs and VSMCs led to reduced GAG contents in cells and attenuated the interaction of isolated EVs with VSOPs in relaxation experiments.

Activation of the PI3K/AKT pathway has been shown to be a prerequisite for the proper function of the SLC26A2 transporter [27] and to play a central role in the remodeling of the ECM including ECs and VSMCs [47,48]. This enables the inhibition of AKT to be an interesting target to prevent cardiovascular remodeling [49]. We here demonstrate that the induced upregulation of SLC26A2 by uremic toxins is accompanied by the activation of AKT signaling and the inhibition of AKT-attenuated SLC26A2 protein expression as well as the generation of GAG in cells and isolated EVs. Such stimulating effects of Pi on AKT signaling have also been described earlier, e.g., in lung cells [50,51].

In line with own previous studies [6], we confirmed our in vitro data by comparing plasma-derived EVs from uremic rats with EVs from healthy rats. EVs from uremic rats contained more GAGs and had a stronger effect on VSOP-T1 relaxivity compared to EVs from control animals. This once again confirmed the important function of GAGs for the interaction between EVs and potential ligands like VSOPs and the functional role of the (patho)physiological environment of the host cells.

In conclusion, our data indicated that the pathophysiological condition of uremia impacts not only the cargo load of EVs [6] but also the surface structure like the degree of sulfation of GAGs. This, in turn, could influence the interaction of EVs with ligands such as VSOPs. The results might help for the development of novel EVs, or tissue selective imaging probes.

### Limitations

Our cell culture models were performed under “classical” static culture conditions. Previous data show that physiological surface shear stress impacts, e.g., the structure of the cellular glycocalyx [52]. Whether the culture conditions also influence the structure or the cargo load of EVs needs to be investigated in further studies. However, our data indicate that in vivo-derived EVs from rats with CKD showed comparable effects and GAG contents like our in vitro-derived EVs from uremic toxin-treated cells.

After the lyases treatment of the EVs, the EVs were obtained using the exosome isolation reagent as already used during the initial isolation of the EVs from cell culture supernatants or rat plasma. We cannot exclude that parts of the enzymes or of degraded GAGs co-precipitated alongside the EVs and thereby impacted subsequent EV measurements.

A previous study identified contaminating structures without a limiting membrane in exosome preparations, isolated from biological fluids such as plasma or urine [53]. These structures were found to be 20–100 nm in size, thus showing the same size as exosomes. Those contaminations and a potential influence on biological effects of extracellular vesicles cannot be excluded from our study.

## 4. Materials and Methods

### 4.1. Cell Culture

Human endothelial cells (EC; EA.Hy926 cell line, ATCC^®^ CRL-2922™) and rat aortic vascular smooth muscle cells (VSMC; A7r5 cell line, ATCC^®^ CRL-1444™) were routinely cultured in a standard culture medium which consisted of DMEM (Gibco/Thermo Fisher, Hennigsdorf, Germany) with 862 mg/L L-alanyl-L-glutamine, 1.0 g/l glucose, 50 μg/mL streptomycin, 50 units/mL penicillin and 10% heat-inactivated foetal bovine serum (FBS, Gibco/Thermo Fisher). 

Chinese hamster epithelial-like ovary cell lines (ATCC^®^ CRL-2242^TM^, CRL-2244^TM^, CRL-61^TM^) were cultured in the DMEM/F-12 (1:1) GlutaMAX^TM^-I medium (Gibco/Thermo Fisher) supplemented with 50 μg/mL streptomycin, 50 unit/mL penicillin and 10% heat-inactivated FBS (Gibco/Thermo Fisher).

FBS was used from the same lot throughout the whole study. Cells were cultured in a humidified atmosphere at 37 °C and 5% CO_2_. Cells were maintained at 70–80% confluence by passaging as needed.

### 4.2. Treatment of Cells with Uremic Toxins

ECs and VSMCs were treated for 7 days in 75 cm^2^ or 125 cm^2^ flasks (NUNC, Roskilde, Denmark) with a standard culture medium containing 3.5 mM NaH_2_PO_4_ (Pi; final concentration) or a mixture (UI) of 20 mM urea (Merck, Darmstadt, Germany) and 375 µM (50 µg/mL) indoxyl sulfate (Sigma-Aldrich, Taufkirchen, Germany). The concentrations of the toxins correspond to concentrations seen during chronic renal failure [54,55]. The medium was replaced every second day. Vehicle-treated cells served as control. 

For a later analysis of cells using qPCR, Western blot or the Blyscan^TM^ assay, the cells were treated in 6-well or 12-well plates (NUNC).

### 4.3. Isolation and Characterization of EVs

After treatment with the uremic toxins, the cells were washed two times with sterile-filtered PBS and cultured for additional 24 h in standard culture medium supplemented with a 5% exosome-depleted FBS (Gibco/Thermo Fisher). The culture medium was then centrifuged at 700× *g* for 5 min to remove cells followed by centrifugation at 2000× *g* for 20 min to remove cellular debris. The remaining supernatants were then concentrated using Vivaspin 20 centrifugal filter devices with 3K NMWL (GE Healthcare, Chicago, IL, USA) at 3220× *g* followed by centrifugation for 30 min at 20,000× *g* to obtain the microvesicle fraction. The supernatant was diluted 1:2 with the Total Exosome Isolation (from cell culture media) Reagent (Invitrogen, Waltham, MA, USA) and incubated on a rotary shaker for 18 h at 4 °C. The exosome fraction was obtained by centrifugation of the solution for 60 min at 10,000× *g*. The pellet was washed twice with ice cold 0.9% NaCl solution (B. Braun Melsungen AG, Melsungen, Germany), resuspended in 100 µL ice cold 0.9% NaCl solution and used immediately or stored for further analyses and experiments at −80 °C. The protein contents of the isolated EV fractions were used as a surrogate marker for the vesicle quantity. They were determined by the Pierce™ BCA™ Protein-Assay (Thermo Fisher).

EVs from rat plasma were isolated with the Total Exosome Isolation (from plasma) Reagent (Invitrogen) without Proteinase K treatment. The pellets were washed, pooled and stored in a NaCl solution as described above. The protein contents of the isolated EV fractions were determined by the Pierce™ BCA™ Protein-Assay. Briefly, the EV samples (dissolved in NaCl) were diluted 1:4 with H_2_O and were directly subjected to the BCA assay according to the manufacturer’s instructions. The different EV populations of interest showed hydrodynamic diameters between 20 and 100 nm and were positive for protein expressions of the exosomal markers CD9, CD63 and CD81 (Supplementary Figure S1).

### 4.4. Synthesis of VSOP

The different steps of VSOP synthesis are described in detail in [56]. Briefly, iron (II) and iron (III) salts were dissolved in water and were precipitated as hydroxides at alkaline conditions. The addition of citric acid lead to solubilization and stabilization of the iron oxide particles by formation of a citrate coat. 

### 4.5. T1 Relaxivity Measurements

We here focused on T1 relaxivity only. The determination of proton T1 relaxation rates was performed at 40 °C and 40 MHz (0.94 T) using an MR spectrometer (Minispec mq 40; Bruker, Karlsruhe, Germany). T1 relaxation rates were determined with an inversion recovery pulse sequence, which is the standard measuring and analysis method of the equipment. 

Measurements were performed in nanopure water containing 0.009% NaCl. For the determination of T1 relaxivities of VSOPs with and without the presence of EVs, VSOPs at a constant concentration of 0.0188 mM were mixed with different EV concentrations (protein contents between 0 and 5.0 µg/mL). Three solutions with different concentrations were measured for each sample. The relaxivity coefficients r1 were obtained by linear fitting of T1 relaxation rates, and values were normalized to the iron concentrations.

### 4.6. Magnetic Particle Spectroscopy (MPS) Measurements

The MPS measurements on VSOP samples were performed using a commercial magnetic particle spectrometer (MPS-3, Bruker, Karlsruhe, Germany), as previously described [36]. MPS detects the non-linear magnetic susceptibility of magnetic nanoparticles such as VSOPs by applying a sinusoidal excitation field *B*_ex_ of 25 mT at a frequency *f*_0_ of 25 kHz to the VSOP sample. Due to the inherent non-linearity of the magnetization curve, the measured response of the VSOP contains odd multiples of *f*_0_, i.e., higher harmonics. In this study, we used the concentration-independent ratio of the fifth and third harmonic, *A*_5_/*A*_3_, which represents the shape of the spectrum, to assess changes in the dynamic magnetization behavior of VSOPs due to interaction with EVs.

MPS measurements were performed in a standard operation mode. Briefly, the interactions between VSOPs and EVs were investigated by in situ real-time MPS measurements. Initially, 125 µg/mL EVs, diluted in a 75 μL NaCl solution, were assembled in a glass tube (Bruker NMS PC 7.5). After placing the glass tube into the MPS pick-up coil, repetitive measurements were started without VSOPs to check the EV preparations for magnetic impurities.

To determine the effects of EVs on the *A*_5_/*A*_3_ ratio of VSOPs, MPS spectra were recorded every 5 s over a time course of 500 s. After 80 s, 75 µL of VSOPs with an iron concentration of 1.34 mmol/L were added to the EVs. As a control, the same procedure was performed without EVs. 

### 4.7. Determination of GAG Contents in Cells and EVs

GAG contents in cells and EVs were determined using the Blyscan^TM^ Sulfated Glycosaminoglycan Assay (Biocolor, Carrickfergus, UK). Cells grown in 6-well plates were washed with PBS and 1.5 mL of the papain protein digestion solution was added. After 10 min, the mixture was transferred to a 2.0 mL tube and the tubes were incubated for 3 h at 65 °C in a water bath with regular shaking every 30 min. For the determination of GAGs in isolated EVs, the EV pellets in 1.5 mL tubes were mixed with 1.0 mL of the papain protein digestion solution. After thorough mixing, the tubes were incubated in the water bath. After 3 h, the papain solutions were centrifuged for 10 min at 10,000× *g* and the supernatants were stored for further analyses at −20 °C.

As a prerequisite for the normalization of measured GAG contents, the amount of double-stranded DNA (dsDNA) in the samples was determined in the supernatants using the Quant-iT Picogreen dsDNA Assay-Kit (Invitrogen, #P11496) as described [57].

For the quantification of GAGs, 100 µL (for EV) or 50 µL (for cells) of the supernatants were mixed with the Blyscan^TM^-dye reagent and the amounts of GAGs were quantified photometrically according to the manufacturer’s protocol. Finally, the data sets were normalized to the dsDNA contents of the samples.

### 4.8. Incubation of EVs with the Lyases Heparinase III and Chondroitinase ABC

For the digestion of surface-associated GAGs, the isolated EVs (125 µg/mL) were incubated with 0.25 sigma units/mL chondroitinase ABC (chon ABC, Sigma, #C3667) or heparinase III (hep III, Sigma, #H8991) alone or as a 1:1 mixture of both enzymes for 1 h at 37 °C in a water bath. The final reaction volume was 100 µL. To obtain the EVs from the solution, the same procedure as for isolating EVs from cell culture supernatants was used [6].

### 4.9. Transfection of Cells with siRNA

By using the HiPerFect Transfection Reagent (Qiagen, Hilden, Germany), VSMCs were transfected with SLC26A2-specific siRNA (Silencer^®^, #AM16708, Thermo Fisher) or a negative control (Silencer™ Negative Control No. 1 siRNA, #AM4611, Thermo Fisher). Cells with a ~75% confluence were treated with a 2 µL siRNA and a 10 µL transfection reagent in a 2 mL standard culture medium for 48 h followed by a 24 h incubation without siRNA and transfection reagent. Cells were then subjected to Western blot and qPCR analysis or were incubated for additional 24 h with a standard culture medium containing a 5% exosome-depleted FBS (Gibco/Thermo Fisher) to isolate EVs from supernatants as described [6].

### 4.10. Western Blot Analyses

Treated cells or pellets of isolated EVs were lysed with a 100 μL lysis buffer and protein concentrations were determined using the Pierce™ Rapid Gold BCA Protein-Assay-Kit (Thermo Fisher). Equivalent amounts (10–50 μg) of protein were subjected to 10% Mini-PROTEAN^®^ TGX™ Precast Protein Gels (Bio-Rad, Feldkirchen, Germany). Blotting of gels was performed using a Mini Trans-Blot^®^ Cell (Bio-Rad). All primary antibodies used are given in Table 2. Bound antibodies on the membranes were visualized using anti-mouse or anti-rabbit WesternBreeze^®^ Chromogenic Immunodetection System Kits (Thermo Fisher). Band densities were quantified using Image J software (version 1.50i; National Institutes of Health, Bethesda, MA, USA).

### 4.11. Inhibition of AKT-Signaling in the Host Cells

The phosphatidylinositol 3 kinase (PI3K)/AKT inhibitor LY294002 (Ly29, Cell signalling) was applied at a concentration of 20 µM to block the uremic toxin-induced phosphorylation of AKT, the toxin-induced expression of SLC26A2 in EC and VSMC, and the toxin-induced generation of GAGs in both cell types. Ly29 was dissolved in DMSO. The maximum DMSO concentration in the experiments was 0.05%. For Western blot experiments, the cells were pre-incubated with 20 µM Ly29 for 10 min prior to the respective treatment with uremic toxins. For longer experiments, the cells were treated prior to every planned medium change (every second day) for 10 min with Ly29.

### 4.12. Rat Model of Chronic Kidney Diseases (CKD)

CKD in rats was induced by adenine-feeding as described [6]. In brief, 9–10-week-old male Wistar rats (Charles River, Écully, France) were fed with adenine (0.3%; Altromin, Lage, Germany) for 4 weeks followed by feeding a high-phosphate diet (1.4% vs. 0.5%) for 16 weeks. Blood samples were collected at the end of the experiments and plasma samples were stored at −20 °C. 

### 4.13. Manipulation of GAG Contents in Host Cells

To artificially decrease GAG contents in host cells, the cells were treated with 15 µM genistein (Sigma) for 3 d. After the treatments, the cells were incubated for 24 h with standard culture medium containing 5% exosome-depleted FBS (Thermo Fisher) to isolate EVs from supernatants as described [6].

### 4.14. HS and CS/DS Disaccharide Analysis of EVs

EV pellets were resuspended in a 50 mM Tris/10 mM CaCl_2_ pH 7.6. A pronase enzyme was added up to a concentration of 10 mg/mL and then the samples were incubated at 37 °C for 18 h. After incubation, 250 U of benzonase in a 2 mM MgCl_2_ were added and incubated in the mixture for 4 h at 37 °C. Afterwards, GAGs were enriched using manual anion exchange chromatography with Q-sepharose beads. Self-packed columns were equilibrated with 20 mM NaOAc/100 mM NaCl pH 5.0 before acidified samples were applied. After thoroughly washing with the equilibration buffer, elution was performed using 20 mM NaOAc/1 M NaCl pH 5.0. After complete elution, desalting of samples was carried out using cold, salt-saturated ethanol precipitation. The samples were stored in ethanol and incubated over night at −20 °C, followed by centrifugation at 4 °C at 21,000× *g* for 20 min. The supernatant was removed, and pellets were dried completely. Pelleted GAGs were resuspended in 20 mM Tris/5 mM CaCl_2_/200 mM NaCl pH 7.0 and incubated at 30 °C. A heparinase cocktail (10 mU for each heparinase I, II and III) was added for heparin disaccharide analysis; for chondroitin sulfate disaccharide analysis, chondroitinase ABC (10 mU) was added. The total volume of each reaction was 40 µL. The mixtures were incubated overnight at 30 °C. After incubation, enzymes were inactivated by heat and samples were centrifuged at 21,000× *g* for 10 min at 4° C. The supernatants were transferred and freeze dried. A small volume of a 0.1 M AMAC solution in acetic acid/DMSO (3:17 *vol*/*vol*) was added to the pellets. Dissolved samples were incubated for 15 min in the dark at room temperature. After incubation, an equal volume of 1 M sodium–cyanoborohydride was added. The samples were incubated for 3 h at 45 °C. After incubation, the samples were centrifuged at 21,000× *g* at 4 °C for 15 min. The supernatants were collected and freeze dried. Pure acetone (500 µL) was added to lyophilized samples. After centrifugation at 21,000× *g*, 4 °C, 15 min, the supernatants were removed and pellets were washed with acetone again. Precipitated samples were dried completely, dissolved in a 2% acetonitrile and then loaded onto an Aquity UPLC BEH C18 column (2.1 × 150 mm, 1.7 µm) which was installed in a Knauer Azura UHPLC system. The mobile phase consisted of 150 mM NH_4_OAc pH 5.6 (A) and the eluent was 100% acetonitrile (B). The starting conditions were 97% A/3% B. A gradient from 3% B to 13% B within 20 min was applied. The detection was performed at an excitation wavelength of 425 nm and emission at 520 nm.

### 4.15. PCR Measurements

Relative gene expressions in host cells were determined by qPCR using TaqMan™ assays. A detailed list of all applied TaqMan probes is given in Table 3. Gene expressions in samples relative to controls were determined by using the 2^−∆∆Ct^ method and normalized to the gene expression of the housekeeping gene ribosomal protein L19 (RPL19).

### 4.16. Statistics

Statistics were calculated using GraphPad Prism version 6.01. Data sets were tested for outliers and normal distribution. The in vitro data were analyzed as follows: two treatment groups were compared by using unpaired t tests. Comparisons of three groups were performed by two-way ANOVA and Tukey’s multiple comparisons test. The in vivo data were analyzed by multiple t tests, Mann–Whitney test (two groups), and Kruskal–Wallis test with Dunn’s multiple comparisons test (more than two groups). *p*-values of <0.05 were considered to be statistically significant.

## Figures and Tables

**Table 1:** Function of the genes examined in the study.

Gene	Function	Reference
B3GNT2	Galactosyltransferase which catalyzes the chain elongation process in GAG synthesis	[19]
B4GALT1	Involved in the synthesis and elongation of GAG chains	[20]
CHST15	Encodes for a 6-O-sulfotransferase being involved in the formation of sulfated GalNAc4S6S residues of CS/DS	[21]
CHSY1	Involved in the synthesis and elongation of GAG chains	[22]
EXT1	Involved in the synthesis of HS-chains	[23]
HAS1	Hyaluronan synthase responsible for the synthesis of hyaluronic acid	[24]
HEX A	Involved in the hydrolysis of GAG components	[25]
HEX B	Involved in the hydrolysis of GAG components	[25]
RPL19	Housekeeping gene	[26]
SLC26A2	Sulfate transporter which regulates sulfation of GAGs	[27]
XYLT2	Glycosyltransferase that initiates biosynthesis of GAG chains in proteoglycans including CS, HS and DS	[28]

**Table 2:** Primary antibodies used for Western blot analyses.

Target	Dilution	Company	Product Number
CD9	1:1000	Stemcell^TM^ Technologies (Köln, Germany)	#100-0211
CD63	1:1000	Stemcell^TM^ Technologies	#100-0211
CD81	1:1000	Stemcell^TM^ Technologies	#100-0211
CHST15	1:1000	Thermo Fisher	#100440
GAPDH	1:2000	Thermo Fisher	#MA5-15738
p-p-AKT	1:1000	Cell Signaling (Danvers, MA, USA)	#2965S
p-AKT	1:1000	Cell Signaling	#4691S
SLC26A2	1:1000	Thermo Fisher	#PA576918

**Table 3:** List of applied TaqMan™ probes (Thermo Fisher) in the gene expression experiments.

Gene	Full Name	Assay-ID (Rat)	Assay-ID (Human)
B3GNT2	UDP-GlcNAc:BetaGal Beta-1,3-N-Acetylglucosaminyl-transferase 2	Rn02112835_s1	Hs01935859_s1
B4GALT1	UDP-Gal:betaGlcNAc beta 1,4-galactosyltransferase, polypeptide 1	Rn01764643_m1	Hs00155245_m1
CHST15	Carbohydrate (N-acetylgalactosamine 4-sulfate 6-O) sulfotransferase	Rn00597859_m1	Hs01031067_m1
CHSY1	Chondroitin sulfate synthase 1	Rn01478125_m1	Hs00208704_m1
EXT1	Exostosin Glycosyltransferase 1	Rn00468764_m1	Hs00609162_m1
HAS1	Hyaluronan synthase 1	Rn01455687_g1	HS04398914_m1
HEX A	Hexosaminidase A	Rn01422539_m1	Hs00942655_m1
HEX B	Hexosaminidase B	Rn01493909_m1	Hs01077594_m1
RPL19	Ribosomal Protein L19	Rn00821265_g1	Hs02338565_gH
SLC26A2	Solute Carrier Family 26 Member 2	Rn00589156_m1	Hs00164423_m1
XYLT2	Xylosyltransferase 2	Rn00574186_m1	Hs01048792_m1

## Data Availability

The data that support the findings of this study are available from the corresponding author upon reasonable request.

## Supplementary Materials

The supporting information can be downloaded at: https://www.mdpi.com/article/10.3390/ijms241814253/s1.

## Abbreviations

A5/A3: high harmonic A5/A3 ratio—describes the magnetic behavior of magnetic particles like VSOPs and could be changed by interaction of VSOPs with binding partners; Chon ABC, chondroitinase ABC; CHO, immortalized Chinese hamster ovary cells; CKD, chronic kidney disease; CS, chondroitin sulfate; ECs, endothelial cells; EVs, extracellular vesicles; GAG, sulfated glycosaminoglycans; GalNAc, N-acetyl-D-galactosamine; GlcA, D-glucuronic acid; GlcNAc, *N*-acetyl-d-glucosamine; hep III, heparinase III; HS, heparan sulfate; IdoA, L-iduronic acid; Ly29, Ly294002—PI3K/AKT-inhibitor; Pi, inorganic phosphate; r1, T1 relaxivity—property of a contrast agent like VSOP which is affected by interaction of VSOPs with surrounding binding partners; SLC26A2, solute carrier family 26 member 2—a sulfate transporter in cell membranes; UHPLC, ultra-high-performance liquid chromatography; UI, mixture of the uremic toxins urea and indoxyl sulfate; VSMCs, vascular smooth muscle cells; VSOPs, very small superparamagnetic iron oxide nanoparticles—a contrast agent for magnetic resonance imaging measurements.

## References

[1] Six I., Flissi N., Lenglet G., Louvet L., Kamel S., Gallet M., Massy Z.A., Liabeuf S. (2020). Uremic Toxins and Vascular Dysfunction. Toxins.

[2] Mizobuchi M., Towler D., Slatopolsky E. (2009). Vascular calcification: The killer of patients with chronic kidney disease. J. Am. Soc. Nephrol..

[3] Giussani M., Triulzi T., Sozzi G., Tagliabue E. (2019). Tumor Extracellular Matrix Remodeling: New Perspectives as a Circulating Tool in the Diagnosis and Prognosis of Solid Tumors. Cells.

[4] Lu P., Takai K., Weaver V.M., Werb Z. (2011). Extracellular matrix degradation and remodeling in development and disease. Cold Spring Harb. Perspect. Biol..

[5] Scott J.E. (1992). Supramolecular organization of extracellular matrix glycosaminoglycans, in vitro and in the tissues. Faseb. J..

[6] Freise C., Querfeld U., Ludwig A., Hamm B., Schnorr J., Taupitz M. (2021). Uraemic extracellular vesicles augment osteogenic transdifferentiation of vascular smooth muscle cells via enhanced AKT signalling and PiT-1 expression. J. Cell. Mol. Med..

[7] Andrews A.M., Rizzo V. (2016). Microparticle-Induced Activation of the Vascular Endothelium Requires Caveolin-1/Caveolae. PLoS ONE.

[8] Burger D., Montezano A.C., Nishigaki N., He Y., Carter A., Touyz R.M. (2011). Endothelial microparticle formation by angiotensin II is mediated via Ang II receptor type I/NADPH oxidase/ Rho kinase pathways targeted to lipid rafts. Arterioscler. Thromb. Vasc. Biol..

[9] Chen N.X., Kiattisunthorn K., O’Neill K.D., Chen X., Moorthi R.N., Gattone V.H., Allen M.R., Moe S.M. (2013). Decreased microRNA is involved in the vascular remodeling abnormalities in chronic kidney disease (CKD). PLoS ONE.

[10] Raposo G., Stoorvogel W. (2013). Extracellular vesicles: Exosomes, microvesicles, and friends. J. Cell Biol..

[11] Chauhan S., Danielson S., Clements V., Edwards N., Ostrand-Rosenberg S., Fenselau C. (2017). Surface Glycoproteins of Exosomes Shed by Myeloid-Derived Suppressor Cells Contribute to Function. J. Proteome Res..

[12] Muncie J.M., Weaver V.M. (2018). The Physical and Biochemical Properties of the Extracellular Matrix Regulate Cell Fate. Curr. Top. Dev. Biol..

[13] Golusda L., Kühl A.A., Lehmann M., Dahlke K., Mueller S., Boehm-Sturm P., Saatz J., Traub H., Schnorr J., Freise C. (2022). Visualization of Inflammation in Experimental Colitis by Magnetic Resonance Imaging Using Very Small Superparamagnetic Iron Oxide Particles. Front. Physiol..

[14] Wagner S., Schnorr J., Ludwig A., Stangl V., Ebert M., Hamm B., Taupitz M. (2013). Contrast-enhanced MR imaging of atherosclerosis using citrate-coated superparamagnetic iron oxide nanoparticles: Calcifying microvesicles as imaging target for plaque characterization. Int. J. Nanomed..

[15] Berndt D., Millward J.M., Schnorr J., Taupitz M., Stangl V., Paul F., Wagner S., Wuerfel J.T., Sack I., Ludwig A. (2017). Inflammation-induced brain endothelial activation leads to uptake of electrostatically stabilized iron oxide nanoparticles via sulfated glycosaminoglycans. Nanomedicine.

[16] Bloembergen N., Morgan L.O. (1961). Proton Relaxation Times in Paramagnetic Solutions. Effects of Electron Spin Relaxation. J. Chem. Phys..

[17] Caravan P., Cloutier N.J., Greenfield M.T., McDermid S.A., Dunham S.U., Bulte J.W., Amedio J.C., Looby R.J., Supkowski R.M., Horrocks W.D. (2002). The interaction of MS-325 with human serum albumin and its effect on proton relaxation rates. J. Am. Chem. Soc..

[18] Stetz M.A., Caro J.A., Kotaru S., Yao X., Marques B.S., Valentine K.G., Wand A.J. (2019). Characterization of Internal Protein Dynamics and Conformational Entropy by NMR Relaxation. Methods Enzymol..

[19] Togayachi A., Sato T., Narimatsu H. (2006). Comprehensive enzymatic characterization of glycosyltransferases with a beta3GT or beta4GT motif. Methods Enzymol..

[20] Qasba P.K., Ramakrishnan B., Boeggeman E. (2008). Structure and function of beta -1,4-galactosyltransferase. Curr. Drug Targets.

[21] Ohtake S., Ito Y., Fukuta M., Habuchi O. (2001). Human N-acetylgalactosamine 4-sulfate 6-O-sulfotransferase cDNA is related to human B cell recombination activating gene-associated gene. J. Biol. Chem..

[22] Izumikawa T., Koike T., Shiozawa S., Sugahara K., Tamura J., Kitagawa H. (2008). Identification of chondroitin sulfate glucuronyltransferase as chondroitin synthase-3 involved in chondroitin polymerization: Chondroitin polymerization is achieved by multiple enzyme complexes consisting of chondroitin synthase family members. J. Biol. Chem..

[23] Sugahara K., Kitagawa H. (2000). Recent advances in the study of the biosynthesis and functions of sulfated glycosaminoglycans. Curr. Opin. Struct. Biol..

[24] Weigel P.H., Hascall V.C., Tammi M. (1997). Hyaluronan synthases. J. Biol. Chem..

[25] Norflus F., Yamanaka S., Proia R.L. (1996). Promoters for the human beta-hexosaminidase genes, HEXA and HEXB. DNA Cell Biol..

[26] Fiddler J.L., Clarke S.L. (2021). Evaluation of candidate reference genes for quantitative real-time PCR analysis in a male rat model of dietary iron deficiency. Genes Nutr..

[27] Park M., Ohana E., Choi S.Y., Lee M.S., Park J.H., Muallem S. (2014). Multiple roles of the SO4(^2−^)/Cl^−^/OH^−^ exchanger protein Slc26a2 in chondrocyte functions. J. Biol. Chem..

[28] Götting C., Kuhn J., Zahn R., Brinkmann T., Kleesiek K. (2000). Molecular Cloning and Expression of Human UDP-d-Xylose:Proteoglycan Core Protein β-d-Xylosyltransferase and its First Isoform XT-II. J. Mol. Biol..

[29] Afroz R., Cao Y., Rostam M.A., Ta H., Xu S., Zheng W., Osman N., Kamato D., Little P.J. (2018). Signalling pathways regulating galactosaminoglycan synthesis and structure in vascular smooth muscle: Implications for lipoprotein binding and atherosclerosis. Pharmacol. Ther..

[30] Ramakrishnan B., Boeggeman E., Ramasamy V., Qasba P.K. (2004). Structure and catalytic cycle of beta-1,4-galactosyltransferase. Curr. Opin. Struct. Biol..

[31] Piotrowska E., Jakóbkiewicz-Banecka J., Barańska S., Tylki-Szymańska A., Czartoryska B., Wegrzyn A., Wegrzyn G. (2006). Genistein-mediated inhibition of glycosaminoglycan synthesis as a basis for gene expression-targeted isoflavone therapy for mucopolysaccharidoses. Eur. J. Hum. Genet..

[32] Ernst S., Langer R., Cooney C.L., Sasisekharan R. (1995). Enzymatic degradation of glycosaminoglycans. Crit. Rev. Biochem. Mol. Biol..

[33] Taupitz M., Stolzenburg N., Ebert M., Schnorr J., Hauptmann R., Kratz H., Hamm B., Wagner S. (2013). Gadolinium-containing magnetic resonance contrast media: Investigation on the possible transchelation of Gd³⁺ to the glycosaminoglycan heparin. Contrast Media Mol. Imaging.

[34] Ludwig A., Poller W.C., Westphal K., Minkwitz S., Lättig-Tünnemann G., Metzkow S., Stangl K., Baumann G., Taupitz M., Wagner S. (2013). Rapid binding of electrostatically stabilized iron oxide nanoparticles to THP-1 monocytic cells via interaction with glycosaminoglycans. Basic. Res. Cardiol..

[35] Mastarone D.J., Harrison V.S., Eckermann A.L., Parigi G., Luchinat C., Meade T.J. (2011). A modular system for the synthesis of multiplexed magnetic resonance probes. J. Am. Chem. Soc..

[36] Poller W.C., Löwa N., Wiekhorst F., Taupitz M., Wagner S., Möller K., Baumann G., Stangl V., Trahms L., Ludwig A. (2016). Magnetic Particle Spectroscopy Reveals Dynamic Changes in the Magnetic Behavior of Very Small Superparamagnetic Iron Oxide Nanoparticles During Cellular Uptake and Enables Determination of Cell-Labeling Efficacy. J. Biomed. Nanotechnol..

[37] Chacko B.K., Scott D.W., Chandler R.T., Patel R.P. (2011). Endothelial surface N-glycans mediate monocyte adhesion and are targets for anti-inflammatory effects of peroxisome proliferator-activated receptor γ ligands. J. Biol. Chem..

[38] Mohamed R., Dayati P., Mehr R.N., Kamato D., Seif F., Babaahmadi-Rezaei H., Little P.J. (2019). Transforming growth factor-β1 mediated CHST11 and CHSY1 mRNA expression is ROS dependent in vascular smooth muscle cells. J. Cell Commun. Signal.

[39] Piva M.B., Suarez E.R., Melo C.M., Cavalheiro R.P., Nader H.B., Pinhal M.A. (2015). Glycosaminoglycans affect heparanase location in CHO cell lines. Glycobiology.

[40] Brézillon S., Untereiner V., Mohamed H.T., Hodin J., Chatron-Colliet A., Maquart F.X., Sockalingum G.D. (2017). Probing glycosaminoglycan spectral signatures in live cells and their conditioned media by Raman microspectroscopy. Analyst.

[41] Theocharis A.D., Theocharis D.A. (2002). High-performance capillary electrophoretic analysis of hyaluronan and galactosaminoglycan-disaccharides in gastrointestinal carcinomas. Differential disaccharide composition as a possible tool-indicator for malignancies. Biomed. Chromatogr..

[42] Pothacharoen P., Siriaunkgul S., Ong-Chai S., Supabandhu J., Kumja P., Wanaphirak C., Sugahara K., Hardingham T., Kongtawelert P. (2006). Raised serum chondroitin sulfate epitope level in ovarian epithelial cancer. J. Biochem..

[43] ten Dam G.B., van de Westerlo E.M., Purushothaman A., Stan R.V., Bulten J., Sweep F.C., Massuger L.F., Sugahara K., van Kuppevelt T.H. (2007). Antibody GD3G7 selected against embryonic glycosaminoglycans defines chondroitin sulfate-E domains highly up-regulated in ovarian cancer and involved in vascular endothelial growth factor binding. Am. J. Pathol..

[44] Krog M., Ejerblad S., Johansson H. (1984). The aortic content of glycosaminoglycans, hydroxyproline and calcium in experimental uraemia with special reference to parathyroidectomy and vitamin-D treatment. Scand. J. Urol. Nephrol..

[45] Corsi A., Riminucci M., Fisher L.W., Bianco P. (2001). Achondrogenesis type IB: Agenesis of cartilage interterritorial matrix as the link between gene defect and pathological skeletal phenotype. Arch. Pathol. Lab. Med..

[46] Dawson P.A., Markovich D. (2005). Pathogenetics of the human SLC26 transporters. Curr. Med. Chem..

[47] de Sousa Mesquita A.P., de Araújo Lopes S., Pernambuco Filho P.C.A., Nader H.B., Lopes C.C. (2017). Acquisition of anoikis resistance promotes alterations in the Ras/ERK and PI3K/Akt signaling pathways and matrix remodeling in endothelial cells. Apoptosis.

[48] Toyoda S., Shin J., Fukuhara A., Otsuki M., Shimomura I. (2022). Transforming growth factor β1 signaling links extracellular matrix remodeling to intracellular lipogenesis upon physiological feeding events. J. Biol. Chem..

[49] Li X.G., Wang Y.B. (2019). SRPK1 gene silencing promotes vascular smooth muscle cell proliferation and vascular remodeling via inhibition of the PI3K/Akt signaling pathway in a rat model of intracranial aneurysms. CNS Neurosci. Ther..

[50] Chang S.H., Yu K.N., Lee Y.S., An G.H., Beck G.R., Colburn N.H., Lee K.H., Cho M.H. (2006). Elevated inorganic phosphate stimulates Akt-ERK1/2-Mnk1 signaling in human lung cells. Am. J. Respir. Cell Mol. Biol..

[51] Jin H., Xu C.X., Lim H.T., Park S.J., Shin J.Y., Chung Y.S., Park S.C., Chang S.H., Youn H.J., Lee K.H. (2009). High dietary inorganic phosphate increases lung tumorigenesis and alters Akt signaling. Am. J. Respir. Crit. Care Med..

[52] Lindner M., Laporte A., Elomaa L., Lee-Thedieck C., Olmer R., Weinhart M. (2022). Flow-induced glycocalyx formation and cell alignment of HUVECs compared to iPSC-derived ECs for tissue engineering applications. Front. Cell Dev. Biol..

[53] Grigor’eva A.E., Dyrkheeva N.S., Bryzgunova O.E., Tamkovich S.N., Chelobanov B.P., Ryabchikova E.I. (2017). Contamination of exosome preparations, isolated from biological fluids. Biomed. Khim.

[54] D’Apolito M., Du X., Pisanelli D., Pettoello-Mantovani M., Campanozzi A., Giacco F., Maffione A.B., Colia A.L., Brownlee M., Giardino I. (2015). Urea-induced ROS cause endothelial dysfunction in chronic renal failure. Atherosclerosis.

[55] Vanholder R., De Smet R., Glorieux G., Argilés A., Baurmeister U., Brunet P., Clark W., Cohen G., De Deyn P.P., Deppisch R. (2003). Review on uremic toxins: Classification, concentration, and interindividual variability. Kidney Int..

[56] de Schellenberger A.A., Hauptmann R., Millward J.M., Schellenberger E., Kobayashi Y., Taupitz M., Infante-Duarte C., Schnorr J., Wagner S. (2017). Synthesis of europium-doped VSOP, customized enhancer solution and improved microscopy fluorescence methodology for unambiguous histological detection. J. Nanobiotechnol..

[57] Farhat Y. PicoGreen Cell Proliferation Assay Protocol. http://protocol-place.com.

