# Supplementary material for: Uremic Toxin-Induced Exosome-like Extracellular Vesicles Contain Enhanced Levels of Sulfated Glycosaminoglycans which Facilitate the Interaction with Very Small Superparamagnetic Iron Oxide Particles
Source: Int J Mol Sci. 2023 Sep 18;24(18):14253. doi: 10.3390/ijms241814253 (PMC10532171; doi:10.3390/ijms241814253)
Supplement: Supplementary file 1 [file ijms-24-14253-s001.zip › ijms-2584524-supplementary.pdf]

Supplemental figures

Figure S1

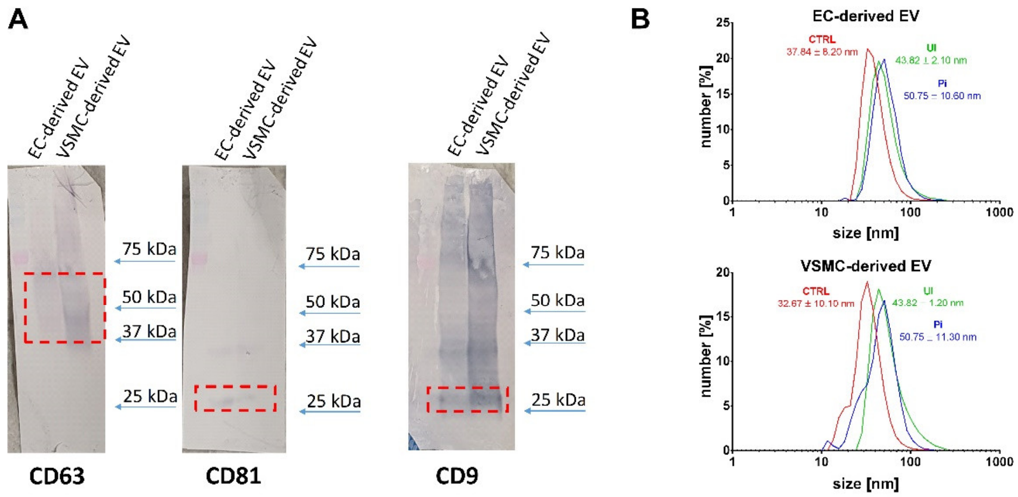

Supplemental Fig. S1: Protein expression of exosomal markers in EV and size determination of EV. (**A**) Isolated EV from EC and VSMC were analyzed by western-blot regarding the protein expression of typical exosomal marker proteins. Shown are representative blots of two independent experiments. (**B**) EC and VSMC were treated with the uremic toxins Pi or UI or left untreated. The hydrodynamic diameters of isolated EV were determined by dynamic light scattering measurements. The measurements were conducted at 23°C using a Zetasizer Nano ZS (Malvern Instruments Ltd., UK), operated at 633 nm and backscattering at 173°. Light scattering was recorded for 10 x 12s with 8 replicate measurements for each sample. Shown are results from one out of three independent experiments. CTRL, control; EC, endothelial cells; *EV, extracellular vesicles; Pi, inorganic phosphate; sGAG, sulfated glycosaminoglycans; UI, mixture of urea and indoxylsulfate; VSMC, vascular smooth muscle cells*

Figure S2

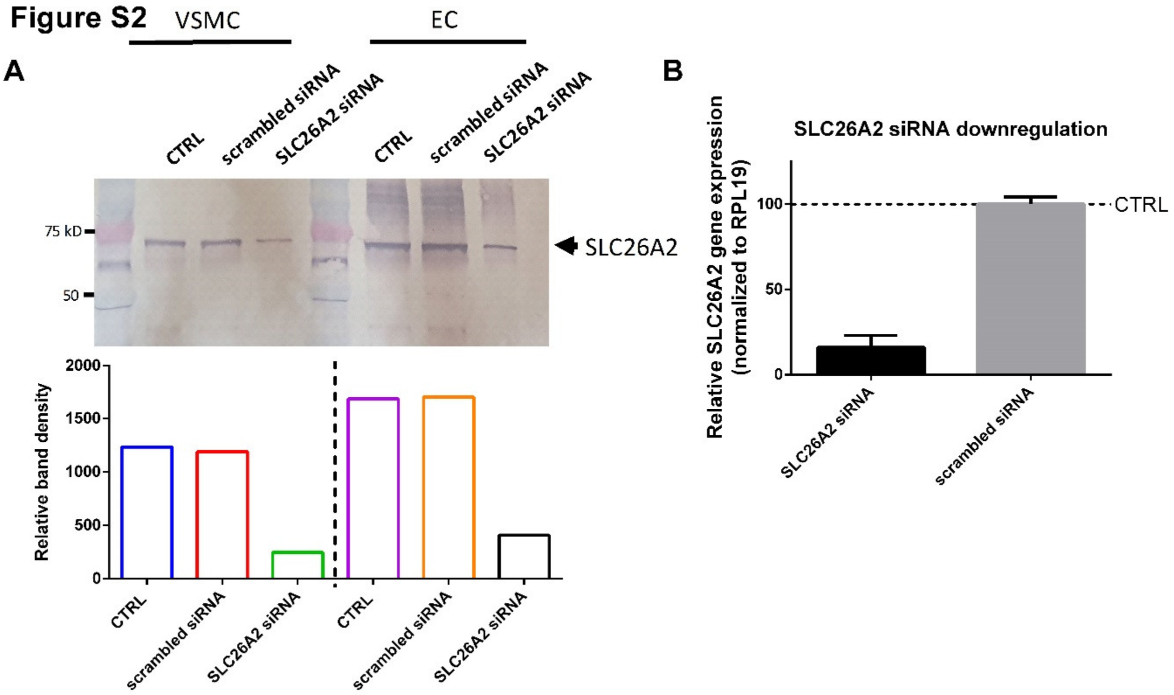

Supplemental Figure S2: Effects of SLC26A2-specific and a scrambled control siRNA on protein expression of SLC26A2. EC and VSMC were treated with a scrambled control siRNA or a SLC26A2-specific siRNA. (**A**) Effects of the treatments on SLC26A2-protein expression were determined by western-blot. Shown is a representative blot out of two independent experiments. (**B**) Relative gene expression of SLC26A2 was determined by qPCR. Shown are means±SD from two indepedent experimets. *CTRL, control; EC, endothelial cells; SLC26A2, a sulfate specific transporter protein; VSMC, vascular smooth muscle cells*

Figure S3

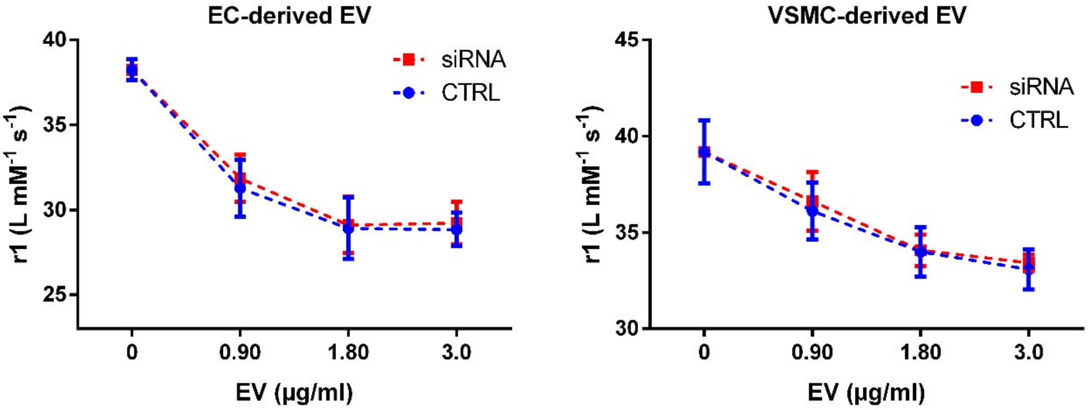

Supplemental Fig. S3: EV from cells treated with the SLC26A2-specific siRNA in the absence of uremic toxins does not influence the T1-relaxivity of VSOP. EC and VSMC were treated with the SLC26A2-specific siRNA or left untreated. EV were isolated from culture supernatants of EC or VSMC and were mixed with VSOP. T1-relaxivites (r1) of VSOP were determined by linear fitting of T1-relaxation rates in relation to VSOP concentrations. Shown are means ± SD (n=2). CTRL, control; EC, endothelial cells; *EV, extracellular vesicles; VSMC, vascular smooth muscle cells*
